# IL-17A weakens the antitumor immuity by inhibiting apoptosis of MDSCs in Lewis lung carcinoma bearing mice

**DOI:** 10.18632/oncotarget.13978

**Published:** 2016-12-16

**Authors:** Juan Wang, Yue Zhang, Kai Yin, Peiqi Xu, Jie Tian, Jie Ma, Xinyu Tian, Yungang Wang, Xinyi Tang, Huaxi Xu, Shengjun Wang

**Affiliations:** ^1^ Department of Laboratory Medicine, The Affiliated People's Hospital, Jiangsu University, Zhenjiang, China; ^2^ Institute of Laboratory Medicine, Jiangsu Key Laboratory of Laboratory Medicine, School of Medicine, Jiangsu University, Zhenjiang, China; ^3^ Department of General Surgery, The Affiliated Hospital, Jiangsu University, Zhenjiang, China; ^4^ Department of Laboratory Medicine, Changzhou TCM Hospital, Changzhou, China

**Keywords:** myeloid-derived suppressor cells, interleukin-17, apoptosis, tumor immunology

## Abstract

Myeloid-derived suppressor cells (MDSCs) weaken the antitumor immune response through the inhibition of effector T cell activity and the production of immunosuppressive factors in pathological sites. It is well established that interleukin-17A (IL-17A) has a remarkable role on the promotion of inflammation and tumor formation, and IL-17 has been implicated in the enhancement of immunosuppression of MDSCs, which consequently promotes tumor progression. A detailed study of this relationship remains elusive. In our study, we not only confirmed the promotion of IL-17 on Lewis lung carcinoma (LLC) development but also surprisingly showed that IL-17 could extend the fate and enhance the immunosuppressive effect of MDSCs through activating ERK1/2. Additionally, the effect of IL-17 on MDSCs was reversed, even in tumors by blocking ERK1/2. Interdicting the signaling molecule ERK1/2 could increase the apoptosis of MDSCs and weaken the suppressive activity of MDSCs, so that thereafter, the antitumor immunity could be restored partly. Therefore, these findings offer new insights into the importance of IL-17 and the downstream signaling factor ERK1/2 for MDSCs.

## INTRODUCTION

Immune responses play important roles in preventing tumor development and invasion. Organism immunity defends against inflammation and tumors. It also suppresses the abnormal enhancement of autoimmune responses. However, once the immune response goes through an endless inflammation process that could promote inflammation development, a tumor begins to develop through accelerating angiogenesis and metastasis and subverting immune responses to promote tumor development for cancer-related inflammation [1]. What's more, in the process from inflammation to tumor, myeloid-derived suppressor cells (MDSCs) increase in the peripheral blood, spleens, and tumor sites. MDSCs represent the immature population of myeloid cells, which are composed of macrophages, dendritic cells, and granulocytes [2]. This immature population plays an important role in escaping from organism antitumor immunity by inducing regulatory T cells and restraining the activation and proliferation of effector T cells [3–4]. In mice, MDSCs consist of two major subsets, named M-MDSCs and G-MDSCs, according to the expression of *ly6C* and *ly6G*. Previous reports have suggested a number of cytokines and transcription factors can influence the differentiation, activation, and expansion of MDSCs [5–8].

Interleukin-17 (IL-17) family is composed of six cytokines, IL-17A–F. Among these cytokines, IL-17A ( also termed IL-17) is an inflammatory cytokine secreted by Th17, Tc17, macrophages, and γδ T cells. IL-17 has been examined extensively in several autoimmune diseases as a proinflammatory regulator, such as rheumatoid arthritis and systemic lupus erythematosus [9–11]. The proinflammatory effect of IL-17 is mediated by the IL-17 receptor (IL-17R), which is widely expressed on B cells, T cells, and neutrophils. However, the role of IL-17 is controversial, although IL-17 is protective under certain circumstances. For example, IL-17 is mainly protective at the lung surface and IL-17−/− or IL-17RA−/− mice are susceptible to infection with *Candida albicans*. In addition, the protection of IL-17 to T cells from apoptosis is also an interesting research area [12]. More and more researchers focus on the ability of IL-17 which drives the development of inflammation and tumors. The knowledge about the function of IL-17 in inflammation and tumors has been gained. IL-17 promotes tumor neovascularization in nude mice via induction of IL-6 and MDSCs indirectly or supporting neoplastic growth directly [13]. IL-17 can also promote tumor growth by enhancing angiogenesis in immunocompetent hosts [14]. Abundant evidence indicate that the development of tumors is associated with IL-17 [15]. In addition, recent reports indicated that IL-17 could induce tumor cells to secrete IL-6 and promote tumor growth by STAT3 pathway [16].

With regard to the roles of IL-17 and MDSCs in tumors, He D et al. evaluated the link between IL-17 and MDSCs, and their findings demonstrated that IL-17-mediated responses promote tumor development via the induction of MDSCs accumulating in tumor sites [17]. At the same time, the Ma S group found that the CXCL5 production of tumor cells could be induced by IL-17A, and the infiltration of MDSCs in tumor sites was mediated by CXCL5/CXCR2 [18]. In addition, γδT17 cells promote the accumulation and expansion of MDSCs in human colorectal cancer (CRC) [19]. However, the relationship between IL-17 and MDSCs is unclear. Hou W and colleagues found that IL-17 and IL-6 synergistically promote persistence by inhibiting cellular apoptosis [20]. ERK1/2 is a downstream factor of IL-17 belonging to the p44/p42 MAPK signaling pathway that can activate p90RSK and CREB to induce the expression of BCL-2 [21–24].

In this study, we present a new mechanism by which IL-17 regulates MDSCs, which promotes the development of tumor. When MDSCs were treated with IL-17 *in vitro*, the later apoptosis rate of MDSC decreased and the rate of alive MDSCs increased. We also found that the expression of the anti-apoptotic protein BCL-2 enhanced. The phosphorylation level of ERK1/2 was upregulated after MDSCs treated with IL-17. More importantly, we demonstrated that inhibition of ERK1/2 could reverse the anti-apoptotic effect of IL-17 on MDSCs. In addition, IL-17 could promote tumor progression by inhibiting MDSC apoptosis in Lewis lung carcinoma (LLC) tumor bearing mice.

## RESULTS

### IL-17 inhibits apoptosis of MDSCs

**Figure 1:**
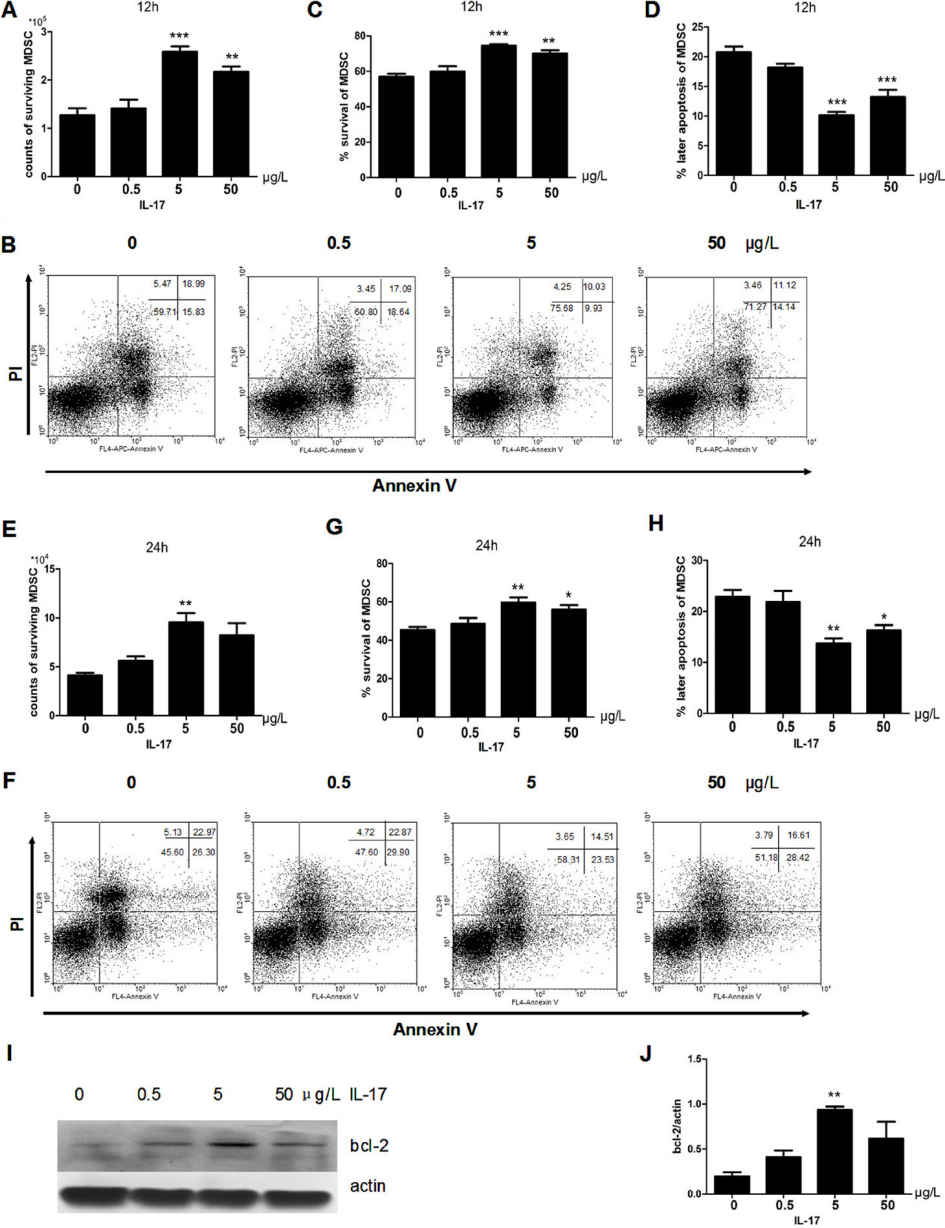
IL-17 inhibits apoptosis of MDSCs MDSCs were isolated from tumor bearing mice and were then treated with different doses of IL-17. (**A**) Counts of surviving MDSCs treated with different doses of IL-17 for 12 h. (**B**) Representative apoptosis analysis of MDSCs treated with IL-17 for 12 h by flow cytometry, plots of cells with ANNEXIN V on the x-axis and propidium iodide on the y-axis. (**C**) Percentage of surviving MDSCs treated with IL-17 for 12 h. (**D**) Percentage of later apoptotic MDSCs treated with IL-17 for 12 h. (**E**) Counts of surviving MDSCs treated with IL-17 for 24 h. (**F**) Representative apoptosis analysis of MDSCs treated with IL-17 for 24 h by flow cytometry (**G**) Percentage of surviving MDSCs treated with IL-17 for 24 h. (**H**) Percentage of later apoptotic MDSCs treated with IL-17 for 24 h. (**I**–**J**) Western blot analysis was used to detect the BCL-2 expression in MDSCs treated with IL-17. BCL-2 level was normalized to β-actin expression. *p < 0.05, **p < 0.01 and ***p < 0.001.

MDSCs from the spleens of tumor bearing mice were stimulated with IL-17. After 12 h, the counts of living MDSCs with IL-17 stimulation were more than the MDSCs with PBS treatment (Figure 1A). We found the survival rate of MDSCs increased with IL-17 treatment, especially at the concentration of 5 μg/L (Figure 1B–1C). However, there was a decrease of the later apoptosis of MDSCs (Figure 1B, 1D) at this optimum concentration with IL-17 treatment. Moreover, we found that this pro-survival and anti-apoptotic effect of IL-17 could last for 24 h. Flow cytometric analysis showed the counts and proportion of living MDSCs increased, and the later apoptosis of MDSCs clearly decreased (Figure 1E–1G). The rate of later apoptosis of MDSCs also decreased after stimulation with IL-17 for 24 h (Figure 1H). Furthermore, as shown in Figure 1I–1J, the expression of BCL-2, a well-known anti-apoptotic protein, was significantly upregulated, especially at the optimum concentration after IL-17 treatment. Thus, IL-17 can promote survival of MDSCs and inhibit apoptosis, involving BCL-2 molecule.

### IL-17 inhibits apoptosis of MDSCs through ERK1/2

**Figure 2:**
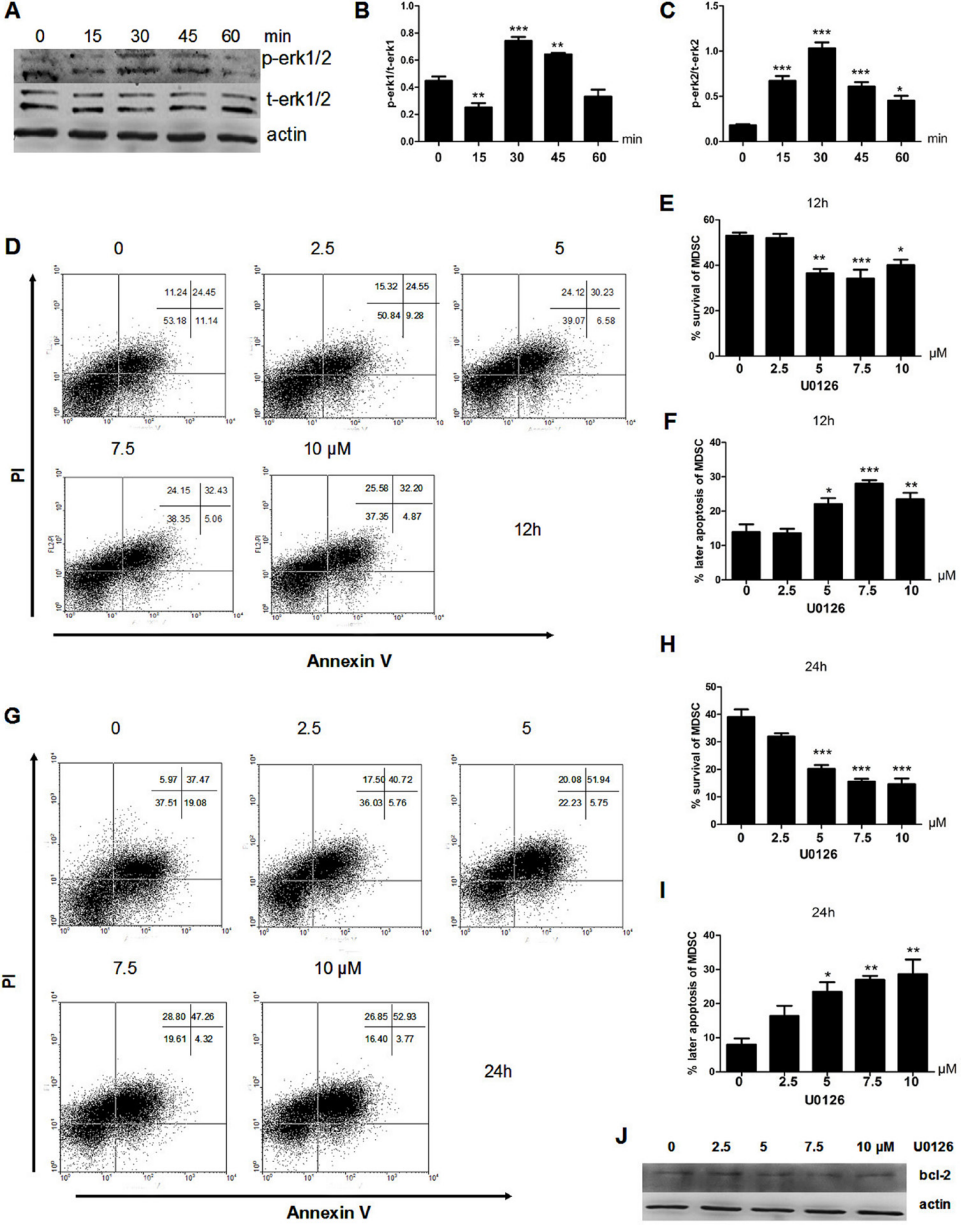
IL-17 inhibits apoptosis of MDSCs through ERK1/2 MDSCs were isolated from tumor bearing mice and then treated with IL-17. (**A**) Western blot was used to confirm the phosphorylation expression of ERK1/2 in MDSCs treated with IL-17. (**B**–**C**) Quantitation of the pERK1/tERK1 (B) or pERK2/tERK2 (C) ratio are shown. (**D**) Representative apoptosis analysis MDSCs after pretreatment with U0126 for 1 h and IL-17 treatment for 12 h by flow cytometry. (**E**–**F**) Percentages of surviving or later apoptosis MDSCs after pretreatment with U0126 for 1 h and IL-17 treatment for 12 h. (**G**) Representative apoptosis analysis MDSCs after pretreatment with U0126 for 1 h and IL-17 treatment for 24 h by flow cytometry. (**H**–**I**) Percentage of surviving and later apoptosis MDSCs after pretreatment with U0126 for 1 h and IL-17 treatment for 24 h. (J) BCL-2 expression after pretreatment with U0126 for 1 h and IL-17 treatment for 24 h. *p < 0.05, ***p < 0.01, and ****p < 0.001.

The results showed that the phosphorylation of ERK1/2 was upregulated after IL-17 treatment and reached a peak at 30 min (Figure 2A) with obvious significance (Figure 2B and 2C). In addition, we found that the anti-apoptotic effect of IL-17 was blocked when pretreated with an ERK1/2 inhibitor U0126 for 12 h before IL-17 treatment (Figure 2D–2F). It is worth mentioning that the influence of IL-17 treatment after 24 h can also be blocked (Figure 2G–2I). In addition, the expression of BCL-2 decreased (Figure 2J) when MDSCs pretreated with U0126 before IL-17 stimulation. Our results demonstrated that IL-17 could inhibit MDSCs apoptosis through ERK1/2, which further induced expression of BCL-2.

### IL-17 promotes LLC tumor development in mice

**Figure 3:**
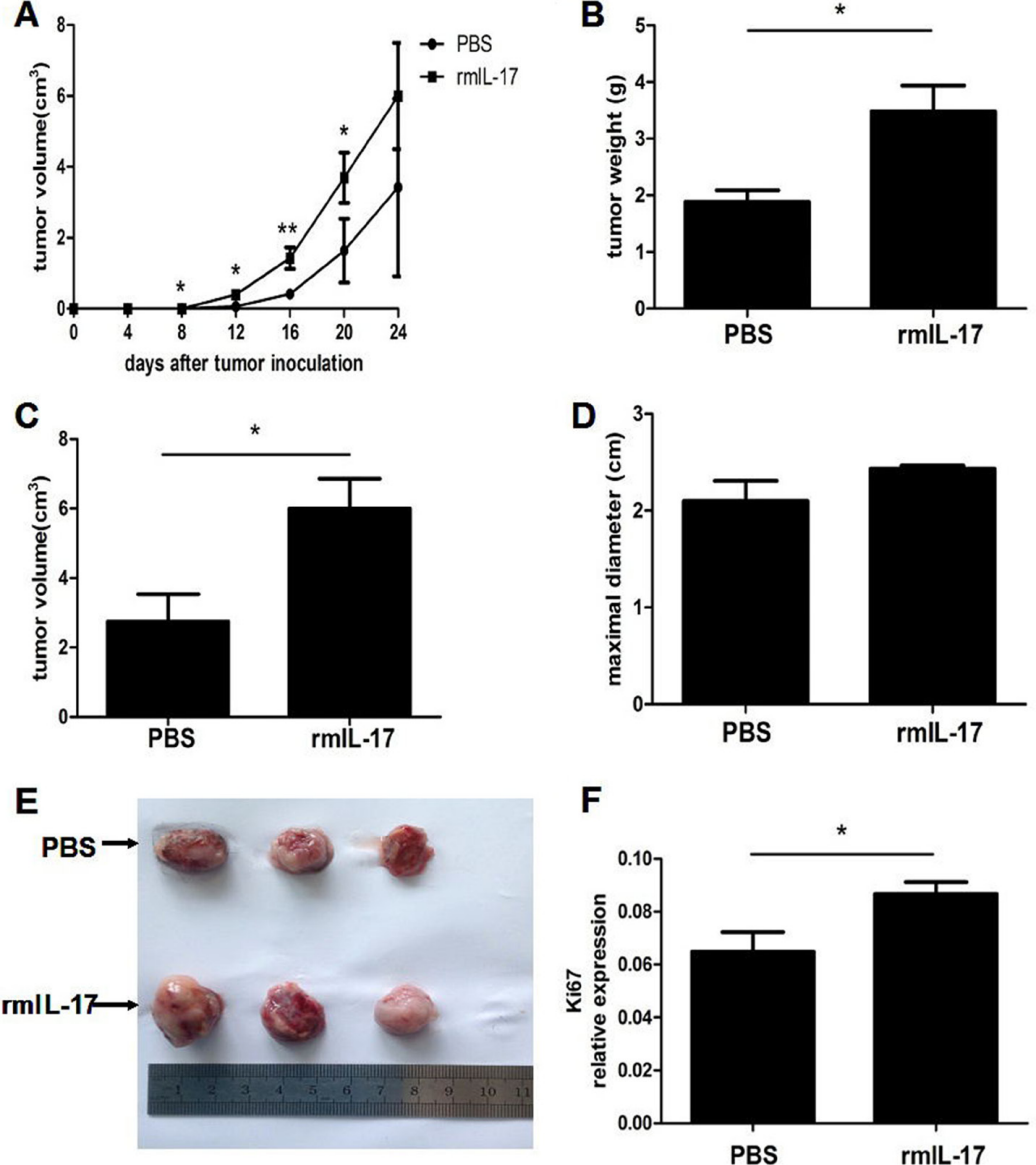
IL-17 promotes LLC tumor development in vivo Lewis lung carcinoma cells were injected subcutaneously into C57BL/6 mice to construct tumor bearing mice. At the same time, the mice were treated with IL-17 intraperitoneally. (**A**) Tumor volume was detected at the indicated time in the IL-17 treated mice or controls. Tumor weight (**B**) tumor volume (**C**) maximal diameter (**D**) and representative tumor morphology (**E**) were shown in the IL-17 treated mice or controls. (**F**) qRT-PCR was used to measure Ki67 mRNA expression in tumor. Data were means ± SD from six different mice. *p < 0.05, **p < 0.01, and ***p < 0.001.

To directly define whether IL-17 can promote LLC tumor development, tumor bearing mice were constructed. As shown in Figure 3A, tumors developed more quickly in the rmIL-17 treated tumor bearing mice compared with the control group. In addition, the tumor volume and weight, as well as maximal diameters were detected. Among these indices, tumor volume and weight increased in rmIL-17 administered tumor bearing mice compared with control group, although there was no significance in the maximal diameter measurements (Figure 3B–3D). Furthermore, visual images also confirmed the pro-tumor effect of IL-17 (Figure 3E). Finally, we detected the expression of Ki67 to reflect the proliferation of tumor cells. And we found that the expression of Ki67 also upregulated in rmIL-17 treated tumor bearing mice (Figure 3F). Based on these results, we confirmed that IL-17 could promote LLC tumor development.

### IL-17 promotes the development of MDSCs in LLC tumor bearing mice

**Figure 4:**
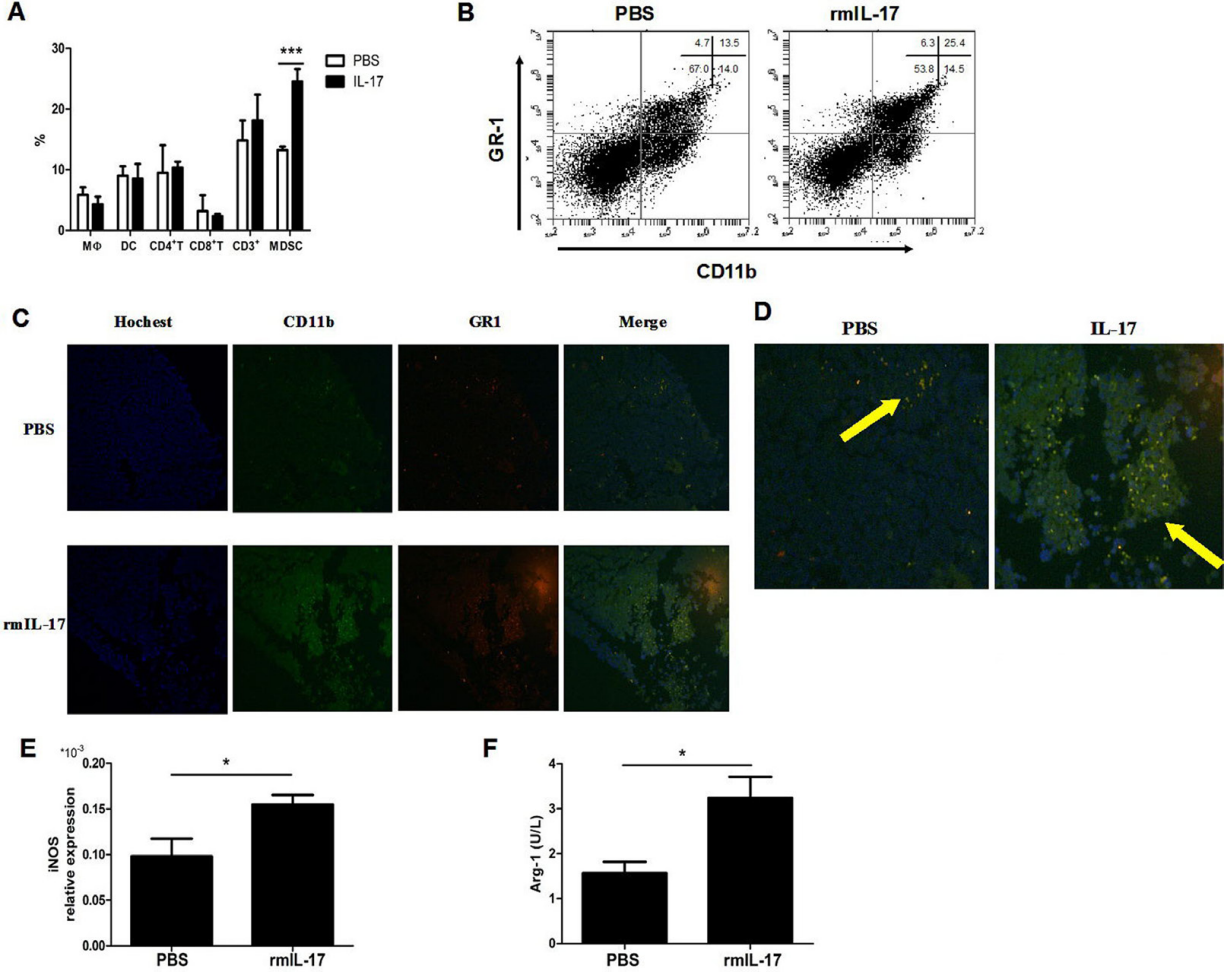
IL-17 promotes the development of MDSCs in LLC tumor bearing mice (**A**) Percentages of macrophage, dendritic cells, CD4^+^ T cells, CD8^+^ T cells, CD3^+^ cells, and MDSCs were quantified by flow cytometry. (**B**) Representative flow cytometry analysis in IL-17 treated mice or controls. (**C**) Counts of MDSCs of tumor in the two groups were shown by IF (original magnification × 40). (**D**) Enlarged image of C (original magnification × 100). The expression of iNOS mRNA (**E**) or the expression of Arg-1 (**F**) were detected in IL-17 treated mice or controls. Data were means ± SD from six different mice. *p < 0.05, **p < 0.01, and ***p < 0.001.

To further demonstrate the critical mechanism by which IL-17 promotes LLC tumor development and to determine whether the effect of IL-17 is associated with MDSCs *in vivo*, we firstly compared the counts of different cell populations infiltrated in the tumors of control group and rmIL-17 treated mice. Except for MDSCs, the counts of other immune cells, including macrophages, dendritic cells, CD4^+^ T cells, and CD8^+^ T cells did not obviously change in the tumor of rmIL-17 treated tumor-bearing mice compared with PBS tumor-bearing mice (Figure 4A and 4B). Further, we found that CD11b^+^Gr1^+^ MDSC increased in tumor tissue from rmIL-17 treated tumor-bearing mice (Figure 4C and 4D). As shown in Figure 4E and 4F, the expression of iNOS and Arg-1 increased in rmIL-17 treated mice. These findings showed that IL-17 could promote the development of MDSCs in LLC tumor bearing mice.

### IL-17 inhibits MDSC apoptosis in LLC tumor bearing mice

**Figure 5:**
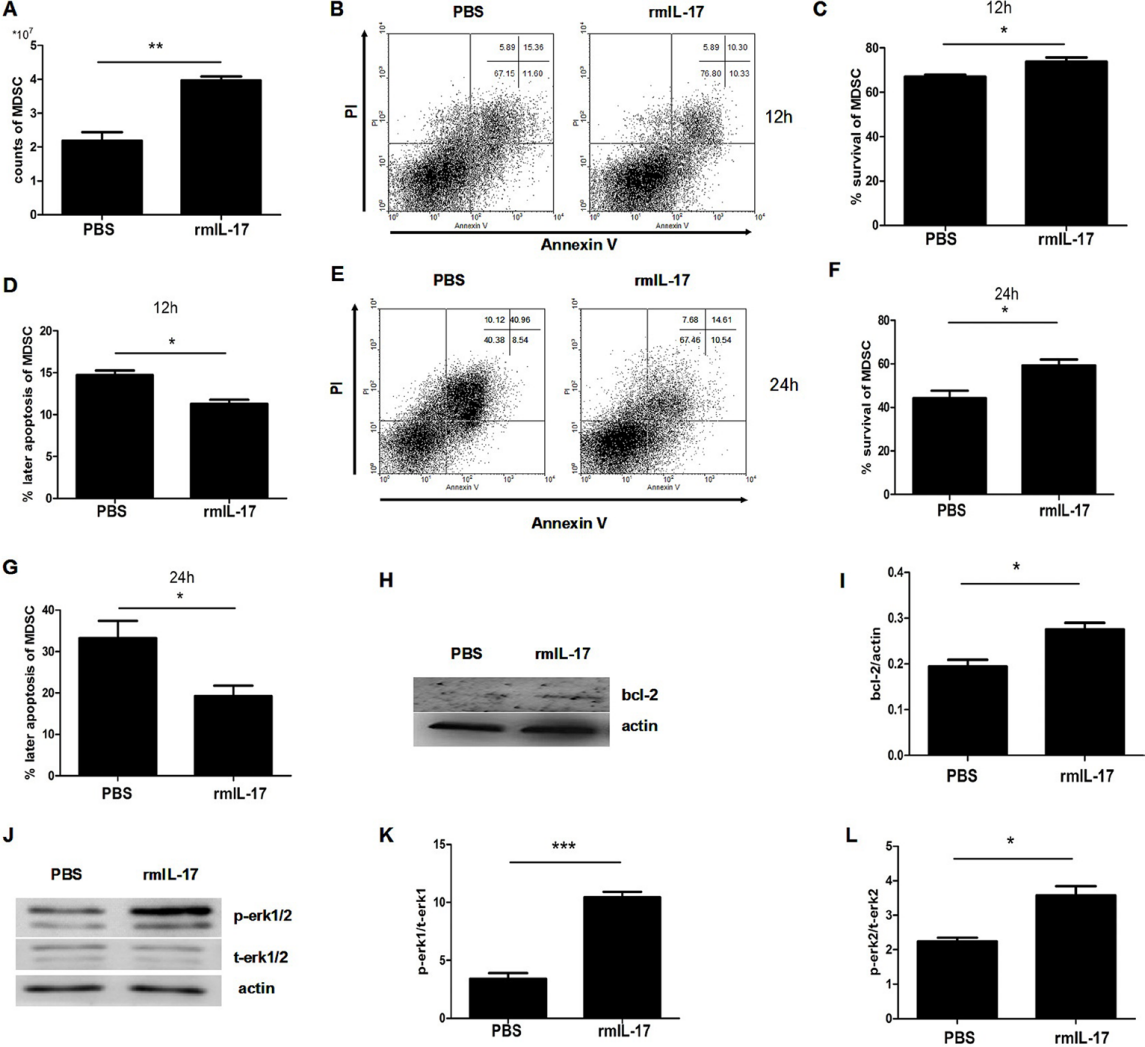
IL-17 inhibits the apoptosis of MDSCs in LLC tumor bearing mice Splenic MDSCs were isolated from IL-17 treated mice or controls and then cultured *in vitro* for 12 h and 24 h. (**A**) Counts of MDSCs of the spleens in IL-17 treated mice or controls. (**B**) Representative apoptosis analysis of MDSCs from IL-17 treated mice or controls at 12 h. (**C**–**D**) Percentage of surviving and later apoptosis MDSCs from IL-17 treated mice or controls at 12 h. (**E**) Representative apoptosis analysis of MDSCs at 24 h. (**F**–**G**) Percentage of surviving and later apoptosis MDSCs from IL-17 treated mice or controls at 24 h. (**H**–**I**) Western blot was used to confirm the expression of BCL-2 in MDSCs. (**J**–**L**) Phosphorylation of ERK1/2 of MDSCs from IL-17 treated mice or controls was detected by western blot. Data were the mean ± SD from six different mice. *p < 0.05, **p < 0.01, and ***p < 0.001.

To examine whether IL-17 could affect the survival of MDSCs, we further tested the influence of IL-17 on MDSCs. First the counts of MDSCs in rmIL-17 treated tumor bearing mice were more than in control mice (Figure 5A). Furthermore, flow cytometric analysis showed the level of survival of MDSCs was higher (Figure 5B) and the rate of later apoptosis of MDSCs was lower in rmIL-17 treated tumor bearing mice (Figure 5C-5D), which could explain the increased accumulation of MDSCs in rmIL-17 tumor bearing mice. Consistent with the results at 12 h, apoptosis of MDSCs in rmIL-17 was also inhibited at 24 h (Figure 5E). The survival of MDSCs significantly increased and later apoptosis of MDSCs markedly decreased in rmIL-17 treated tumor bearing mice (Figure 5F and 5G). In addition, we found the expression of BCL-2 upregulated in rmIL-17 treated tumor bearing mice (Figure 5H and 5I). To examine whether IL-17 inhibited MDSCs apoptosis through ERK1/2 pathway, the phosphorylation of ERK1/2 was detected. As shown in Figure 5J–5L, the phosphorylation of P-ERK1/2 was significantly augmented after rmIL-17 treatment. Our results demonstrate that IL-17 could inhibit MDSC apoptosis in LLC tumor bearing mice.

### IL-17 inhibits MDSC apoptosis through ERK1/2 pathway in tumor bearing mice

**Figure 6:**
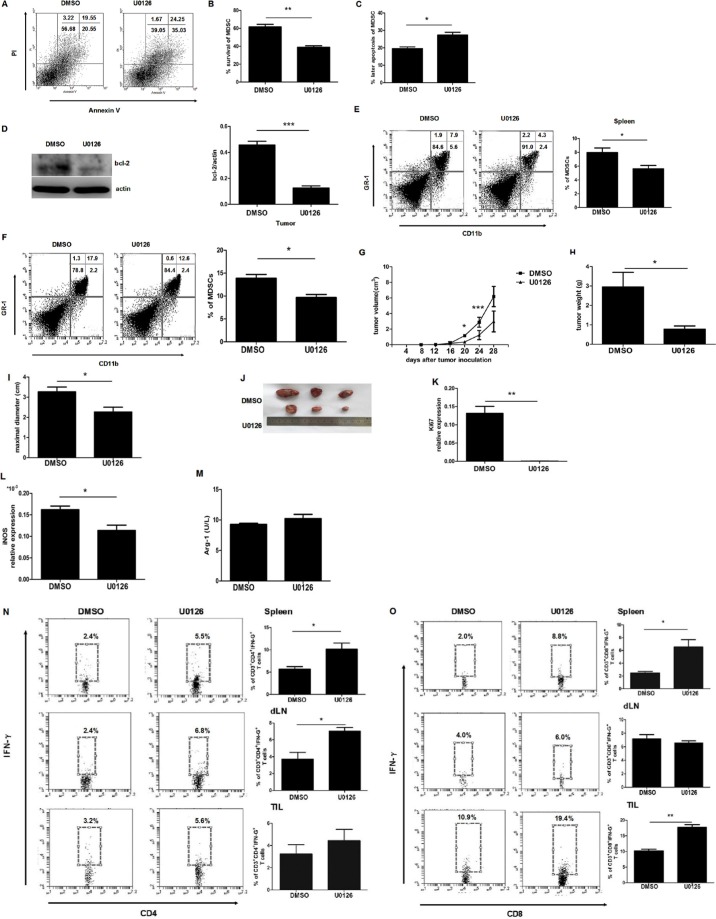
IL-17 inhibits the apoptosis of MDSCs through ERK1/2 in tumor bearing mice Splenic MDSCs were sorted from U0126 or control treated tumor bearing mice. (**A**) Representative apoptosis analysis of MDSCs from U0126 or control treated tumor bearing mice. (**B**–**C**) Percentage of surviving and later apoptosis MDSCs from U0126 or control treated tumor bearing mice. (**D**) BCL-2 expression of MDSCs from U0126 or control treated tumor bearing mice. (**E**–**F**) Percentages of MDSCs in spleens and tumors tissue from U0126 or control treated tumor bearing mice. (**G**) Tumor volume was detected at the indicated times in U0126 or control treated tumor bearing mice. Tumor weight (**H**), maximal diameter (**I**), and representative tumor morphology (**J**) were shown in U0126 or control treated tumor bearing mice. (**K**) Ki67 mRNA level was detected by qRT-PCR in U0126 or control treated tumor bearing mice. The expression of iNOS mRNA (**L**) or the expression of Arg-1 (**M**) were detected in U0126 or control treated tumor bearing mice. The proportions of CD4+IFN-γ+ T cells (**N**) or CD8+ IFN-γ+ T cells (**O**) from the spleen, draining lymph nodes, and tumor tissues were measured by flow cytometry. Data were means ± SD from six different mice. *p < 0.05, **p < 0.01, and ***p < 0.001.

It is well documented that gemcitabine (GEM) can selectively reduce the accumulation of MDSCs in tumor bearing mice and that it does not affect the numbers of T cells. U0126 pretreated MDSCs were tranfered to GEM treated tumor bearing mice. After twice adoptive transfers, we found the apoptotic process of MDSCs from U0126 group was faster than from control group (Figure 6A–6C). Surprisingly, we also observed that U0126 treatment could significantly decrease the percentage of MDSCs in spleens and tumor tissues (Figure 6E and 6F). In addition, tumor development of the U0126 group was slower than the DMSO group (Figure 6G), tumor weight and volume also apparently decreased in the U0126 group (Figure 6H–6J). The expression of Ki67 in the tumors was also obviously downregulated in U0126 group (Figure 6K). Our experiments showed that the expression of iNOS was downregulated in the U0126 group compared to the DMSO group, but the expression of AGR-1 had no change in two groups (Figure 6L and 6M). Then we compared the percentage of CD4^+^IFN-γ^+^ and CD8^+^IFN-γ^+^ cells in spleens, DLNs and tumors in these two groups. We found that U0126 treatment was capable of reinforcing the population of Th1 cells in the spleen and draining lymph nodes (Figure 6N). Meanwhile, the U0126 treatment enhanced the population of Tc1 in the spleen and tumors, especially in tumors (Figure 6O). These findings confirmed our hypothesis that U0126 treatment can shorten the life span of MDSCs, weaken the immunosuppressive effect of MDSCs, and partially strengthen antitumor immune responses.

## DISCUSSION

The IL-17 has become an important research interest since its discovery nearly three decades ago. IL-17 appears to play a controversial role in several diseases. At the lung surface, IL-17 protects against infection [25]. IL-17 also has a dual role in allergies and asthma [26]. Charles’ group found that γδT17 cells exist in lungs of allergic mice, commonly for airway repair [27]. In contrast, a line of evidence suggested that IL-17 damages the self-protective effect of organisms and promotes tumor growth, especially under inflammatory circumstances [28]. IL-17 promotes tumor growth in hepatocellular carcinoma and in human CRC [18–19]. Chang and colleagues reported that Th17 cells play important roles in lung cancer [29]. Wang et al. also reported that IL-17 induces IL-6 production by stromal cells and tumor cells, which express IL-17R. They also found that IL-6 further promotes tumor growth, which is dependent on the STAT3 signaling pathway [30]. What's more, as we known, the major IL-17A-producing cell is Th17 cell which is a branch of CD4^+^T cells. Apart from Th17 cell, several others cell types are considered as sources of IL-17A contain γδT17 cells, CD8^+^T cells, lymph tissue inducers cells(LTi cells), and natural killer T (NKT cells). It is because γδT17 cells and Th17 cells play important roles in lung diseases that we proposed hypothesis that γδT17 cells and Th17 cells could produce IL-17 to promote lung tumor development [25–28].

In the literature, a large amount of knowledge has been provided by studies of the relationship between IL-17 and MDSCs. He et al. reported that IL-17 is required for the infiltration and pro-tumor ability of MDSCs in a lymphoma model. They suggested that IL-17-mediated regulation of MDSCs is a primary mechanism for pro-tumor influences [17]. Furthermore, in human colorectal cancer, γδT17 cells attract tumor chemoattractant PMN-MDSCs and further expand and enhance the survival advantage to promote tumor growth [19]. However, until now, only a few researchers have investigated the effect of IL-17 on the survival of MDSCs. Toh et al. found that IL-17 could extend the survival of fibroblast-like synoviocytes (FLSs) through the regulation of synoviolin expression [31]. Boggio et al. held the same opinion that IL-17 has an anti-apoptotic effect and confirmed that IL-17 can protect T cells from apoptosis [12]. These data further confirmed IL-17 is associated with the apoptosis of cells.

Summarizing the investigations on MDSCs, most have focused on differentiation, induction, activation, expansion, or function [32]. The mechanisms regulating MDSCs turnover and survival are not well understood. However, MDSCs consist of G-MDSCs and M-MDSCs. Despite effect of MDSCs subsets are different, the influence of MDSCs on other cells or organs is by combining effect of two subsets. Our studies provide a novel mechanism for the effect of IL-17 on total MDSCs. An intriguing result was that IL-17 could inhibit MDSC apoptosis in our experiment, and inhibition of the IL-17 anti-apoptotic pathway substantially increased apoptosis of MDSCs. These results are consistent with a report that mentioned IL-17 enhanced the survival advantage of MDSC. In addition, 5FU selectively induced apoptosis of MDSCs by activating caspase-3 and caspase-7 [33]. Furthermore, Chornoguz et al. analyzed MDSC survival and determined that caspase pathways are involved in MDSC apoptosis [34]. The turnover of MDSCs was demonstrated to be regulated by Fas-FasL [35]. ERK1/2, a member of mitogen-activated protein (MAP) kinase and a downstream factor of IL-17 signaling, participated in the receipt of extracellular stimuli, such as growth factor and mitogens. Some researchers reported that oetenpoptin induces expansion of the myeloid progenitor population, such as MDSCs, by activation of the ERK1/2 signaling pathway [36]. In this study, we observed that the phosphorylation of p-ERK1/2 was drastically upregulated in IL-17 treated MDSCs, and the expression of BCL-2 was also being regulated. The anti-apoptotic effect of IL-17 was blocked when MDSCs were treated with ERK1/2 inhibitor. Arg-1 and iNOS are both highly expressed in MDSCs derived from tumor bearing mice [37]. NO generated from iNOS can suppress T cell function and Arg-1 depletes from T cell amino acid L-arginine to inhibit T cell function. In analogy to M1/M2 polarization in macrophages, MDSCs exhibit M2 characteristics, which promotes tumor growth by enhancing ARG-1 activity. MDSCs produce iNOS and consequently produce NO expression. NO prevents proliferation of T cells and render CD8^+^T cells susceptible to FasL-mediated apoptois [38]. It is conceivable that IL-17 can promote the survival of MDSCs and enhance the suppressive activity through ERK1/2 and further be in favor of tumor growth. Taken together, these findings suggest that the IL-17/ERK1/2/BCL-2 pathway plays an important role in the survival and suppressive effects of MDSCs. MDSCs promote tumor growth and confront with immune protection mechanism. Many researchers noted that IL-17 promotes tumor growth by promoting MDSC accumulation and enhancing immunosuppression. We here report for the first time that IL-17 can promote tumor growth through inhibition of MDSC apoptosis, which maybe dependent on ERK1/2 molecule. We here also confirmed the important role of IL-17 in the apoptosis of MDSCs during the tumor development process. These findings further provide evidence that MDSCs promoted tumor development through several pathways and have enriched our knowledge about the association between IL-17 and MDSCs.

## MATERIALS AND METHODS

Mice, cells, and tumor model: male C57BL/6 mice approximately 6–8 weeks old were purchased from Yangzhou University Animal Center. Mouse care and experimental procedures were performed in SPF (specific pathogen-free) conditions. The Lewis lung carcinoma (LLC) cells were obtained from the American Type Culture Collection. Mice were implanted with LLC (2 × 10^6^/mouse) subcutaneously to construct tumor-bearing models. All animal protocols were approved by the Institutional Laboratory Animal Care and Use Committee at Jiangsu University.

Reagents: the LLC cells were cultured in Dulbecco's Modified Eagle Medium and supplemented with 10% FBS and 100 IU/ml penicillin/streptomycin and maintained at 37°C with 5% CO_2_. Recombinant mouse (rm) IL-17 was purchased from Pepro Tech (Rocky Hill, NJ, USA).

Isolation of MDSCs: murine total MDSCs were isolated as previously described [39]. Briefly, splencytes form tumor-bearing mice were isolated using anti-CD11b (BD)conjugated to biotin followed by anti-biotin microbeads (Miltenyi Biotec, Auburn, CA) according to manufacturer's protocol. The purity of CD11b^+^ was greater than 92% and 95% of CD11b^+^ cells were Gr-1^+^CD11b^+^ cells approximately.

Gemcitabine (GEM) treatment and adoptive transfer of MDSCs: Gemcitabine is known as effective anti-tumor drug for treatment of a variety of cancers. It was recently shown to improve anti-tumor response by selectively targeting MDSCs in several preclinical models [40–42]. LLC tumor-bearing mice were received GEM (LC laboratories, Woburn, MA, 200 mg/kg) treatment twice i.p. at 4-day intervals when tumors were 0.5 diameter at day 8–10 after tumor implanted. MDSCs derived from tumor-bearing mice were treated with U0126 (ERK1/2 inhibitor, CST, 10 μM) or DMSO, which is a dilution of U0126 and then were transferred intravenously (5 × 10^6^) twice at one-week intervals.

Reverse transcript PCR and quantitative real-time PCR: total RNA were extracted as described previously [43]. cDNA was synthesized using a random primer and the ReverTraAca^®^ qPCR RT kit (Toyobo, Osaka, Japan). The expression of Ki67 and iNOs was measured with cDNA by qRT-PCR in triplicate using the Bio-Rad SYBR Green Super Mix (Bio-Rad, Hercules, USA), and the relative expression was calculated relative to β-actin according to control cells by the comparative threshold cycle method. The sequences for the primers used were: Ki67: 5′-CAAGGAAGTGTTGGTGGACA-3′ (forward) and 5′-GCAAAGCCCTGGTTCTCAC-3′ (reverse); iNOS: 5′-AACTTGTTTGCAGGCGTCAG-3′ (forward) and 5′-CACATTGCTCAGGGGATGGA-3′ (reverse); and β-actin: 5′-TGGAATCCTGTGGCATCCATGAAAC-3′(forward) and 5′-TAAAACGCAG CTCAGTAACAGTCCG-3′ (reverse).

Western blot analysis: protein extracted from cells was prepared as described previously [44]. Equal amounts of lysates were boiled at 100°C and were then resolved by 12% SDS-PAGE and transferred onto immobilon polyvinylidene difluoride (PVDF) membranes (Millipore, Billerica, MA, USA). Then the membranes were blocked with 5% milk in tris-buffered saline with Tween 20. The specific rabbit antibody against the extracellular signal-regulated kinase (ERK), phosphorylated(p)-ERK1/2 (CST,1:800), BCL-2 (CST,1:500) followed by the secondary HRP-conjugated goat anti-rabbited IgG (CST) were used according to manufacturer's instructions. Proteins were visualized with electrochemiluminescence (ECL) and the relevant blots were quantified by densitometry using the accompanying analysis program (Amercontrol Biosciences, USA).

Flow cytometry analysis: for cell-surface staining, cell samples were stained with fluorescent dye-conjugated mAb against selected markers for 30 minutes on ice. Intracellular cytokine cells were stimulated with PMA (Sigma-Aldrich, St. Louis, MO; 50 ng/mL), ionomycin (eBioscience, San Diego, CA; 1 μg/ml), and monensin (eBioscience, 2 μg/ml) for 5 hours. Then anti-CD3 and anti-CD8 or anti-CD4 (eBioscience) antibodies were used to stain cells, then were fixed, permeabilized, and stained with anti-IFN-γ mAb (eBioscience) according to the Intracellular staining (Invitrogen, Carlsbad, CA) instructions [45].

Assay for cell apoptosis: MDSCs were stimulated with or without rmIL-17 by the indicated concentrations for 12 and 24 h, and then the apoptotic cells were stained with APC-Annexin V (eBioscience) and propidium iodide (eBioscience) and analyzed by FACS, as described previously.

Assay for arginase activity: arginase activity was detected with the QuantiChrom Arginase Assay Kit (BioAssay systems, Hayward, CA) and the arginase activity was computed following the manufacturer's instructions.

Immunofluorescent (IF) staining: formalin-fixed and paraffin-embedded samples were processed for IF staining and the staining was performed by standard protocols using primary anti-CD11b (BD, 1:100), anti-GR-1 (BD, 1:200) at 4°C overnight. Secondary antibodies were FITC-conjugated goat-mouse IgG or PE-conjugated goat-rabbit IgG (Invitrogen). Images were viewed with a fluorescence microscope (Olympus, Japan) and analyzed using the image J software.

*In vivo* experiments: tumor-bearing mice administrated i.p. in triplicate with rmIL-17 or PBS (2.5 μg/mouse) at one week intervals from the day the tumor was implanted. The tumor volume was calculated by the formula V = 1/2a^2^b where “a” was the smaller diameter and “b” was the larger diameter [44]. The tumor weight was determined when the mice were sacrificed. Tumor tissues were cut into small pieces (1–2 mm^3^) and digested with collagenase at 37°C for 2 h. To confirm the effect of ERK1/2 on the anti-apoptotic influence of IL-17 to MDSC *in vivo*, MDSCs were isolated from spleens in other tumor-bearing mice and treated with U0126 (10 μM) and DMSO and then adoptive transfer to tumor-bearing mice occurred after treatment of GEM as described previously.

Statistical analysis: all data were analyzed with Student's *t*-test and presented as the mean ± SD. Data from all experiments were analyzed by GraphPad Prism 5 software and *P* values less than 0.05 were considered statistically significant. *, **, and *** represent statistical significance of *p* < 0.05, *p* < 0.01, and *p* < 0.001, respectively.
