# Correction: IL-17A weakens the antitumor immunity by inhibiting apoptosis of MDSCs in Lewis lung carcinoma bearing mice

**DOI:** 10.18632/oncotarget.28052

**Published:** 2022-02-18

**Authors:** Juan Wang, Yue Zhang, Kai Yin, Peiqi Xu, Jie Tian, Jie Ma, Xinyu Tian, Yungang Wang, Xinyi Tang, Huaxi Xu, Shengjun Wang

**Affiliations:** ^1^ Department of Laboratory Medicine, The Affiliated People’s Hospital, Jiangsu University, Zhenjiang, China; ^2^ Institute of Laboratory Medicine, Jiangsu Key Laboratory of Laboratory Medicine, School of Medicine, Jiangsu University, Zhenjiang, China; ^3^ Department of General Surgery, The Affiliated Hospital, Jiangsu University, Zhenjiang, China; ^4^ Department of Laboratory Medicine, Changzhou TCM Hospital, Changzhou, China; ^*^ These authors contributed equally to this work

**This article has been corrected:** Due to errors during figure assembly, an accidental duplicate image of panel 4 in Figure 1F was inserted in panel 2 of Figure 1F. The corrected Figure 1F, obtained using the original data, is shown below. The authors declare that these corrections do not change the results or conclusions of this paper.

Original article: Oncotarget. 2017; 8:4814–4825. 4814-4825. https://doi.org/10.18632/oncotarget.13978

**Figure 1:**
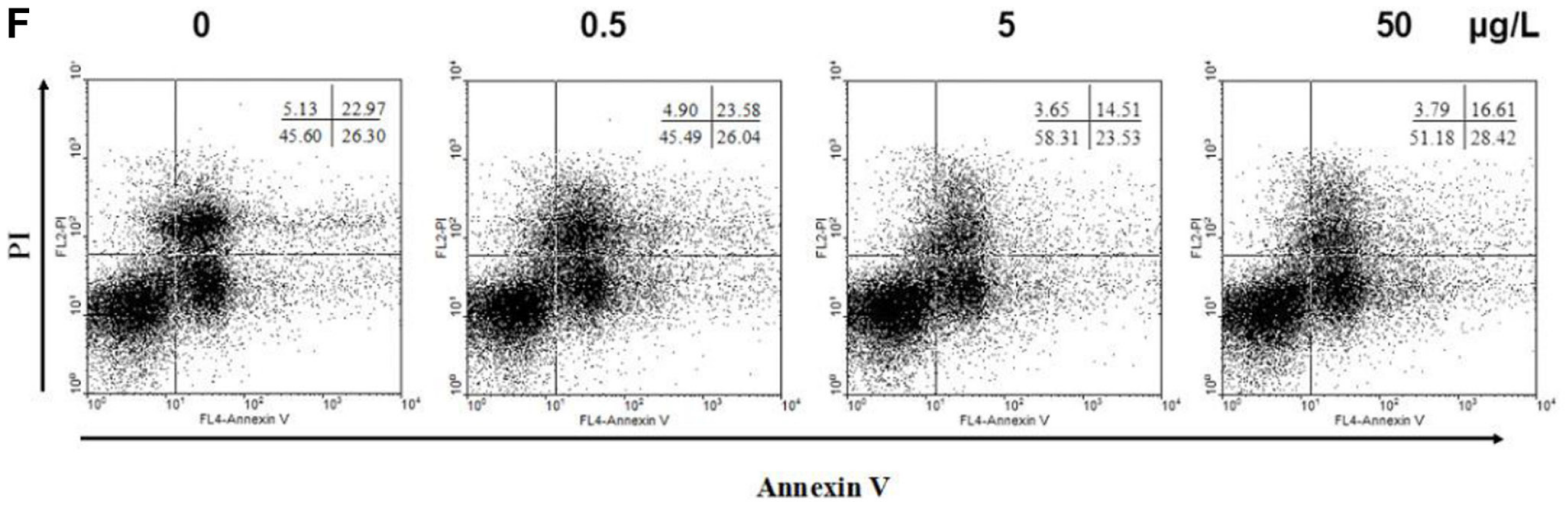
IL-17 inhibits apoptosis of MDSCs. MDSCs were isolated from tumor bearing mice and were then treated with different doses of IL-17. (**F**) Representative apoptosis analysis of MDSCs treated with IL-17 for 24 h by flow cytometry.

